# Treatment of fly ash from power plants using thermal plasma

**DOI:** 10.3762/bjnano.8.105

**Published:** 2017-05-11

**Authors:** Sulaiman Al-Mayman, Ibrahim AlShunaifi, Abdullah Albeladi, Imed Ghiloufi, Saud Binjuwair

**Affiliations:** 1 King Abdulaziz City for Science and Technology (KACST), National Center for Combustion & Plasma Technology, Riyadh, Saudi Arabia; 2 Al Imam Mohammad Ibn Saud Islamic University (IMSIU), College of Sciences, Riyadh, Saudi Arabia

**Keywords:** fly ash, power plant, stabilization/solidification, surface characterization, thermal plasma

## Abstract

Fly ash from power plants is very toxic because it contains heavy metals. In this study fly ash was treated with a thermal plasma. Before their treatment, the fly ash was analyzed by many technics such as X-ray fluorescence, CHN elemental analysis, inductively coupled plasma atomic emission spectroscopy and scanning electron microscopy. With these technics, the composition, the chemical and physical proprieties of fly ash are determined. The results obtained by these analysis show that fly ash is mainly composed of carbon, and it contains also sulfur and metals such as V, Ca, Mg, Na, Fe, Ni, and Rh. The scanning electron microscopy analysis shows that fly ash particles are porous and have very irregular shapes with particle sizes of 20–50 μm. The treatment of fly ash was carried out in a plasma reactor and in two steps. In the first step, fly ash was treated in a pyrolysis/combustion plasma system to reduce the fraction of carbon. In the second step, the product obtained by the combustion of fly ash was vitrified in a plasma furnace. The leaching results show that the fly ash was detoxified by plasma vitrification and the produced slag is amorphous and glassy.

## Introduction

Fly ash is a residue material produced in power plants. This fly ash contains a high level of residual carbon [1], and it contains also transition metals (Fe, Mn, and Co) and alkaline earth metals (Ba, Ca, and Mg). These metals are added to the fuel oils for the suppression of soot or for corrosion control [2–3]. This fly ash is toxic because it contains a high percentage of heavy metals, such as V and Ni. Furthermore, the landfill of fly ash is expensive and causes several environmental problems such as pollution of the soil with organic compounds, leaching of heavy metals and secondary dust generation. Consequently, the treatment of fly ash is an essential process prior to storing it [4].

To treat fly ash many methods have been developed. For example, fly ash was disposed in landfill after being stabilized or immobilized by hydraulic binder [5]. Another technique used to treat the fly ash is thermal plasma. The temperature inside the plasma furnace varied between 4000 and 20000 K. At this range of temperatures, all existing substances will be vitrified, reducing the product mass and converting it to a glassy slag with lower leachability than bottle glass [6–7]. For this reason, thermal plasma is a promising technology for the treatment of fly ash [8–10]. Furthermore plasma technology was used to treat the most toxic waste like radioactive wastes, contaminated hospital wastes, and organohalogen wastes [11].

The first objective of the present study was to characterize the fly ash from power plants using heavy fuel oils in Saudi Arabia. After the characterization, the fly ash will be treated by thermal plasma system built in the laboratory at KACST, which is based on plasma arc technology. The second objective of this investigation was to evaluate the process regarding the following parameters: 1) the percentage of reduction in mass and volume of fly ash, 2) the rate of detoxification (removal of hazardous elements) of fly ash and 3) the stability of the produced slag and the behavior of hazardous materials in leaching tests.

## Experimental

### Thermal plasma system

**Figure 1:**
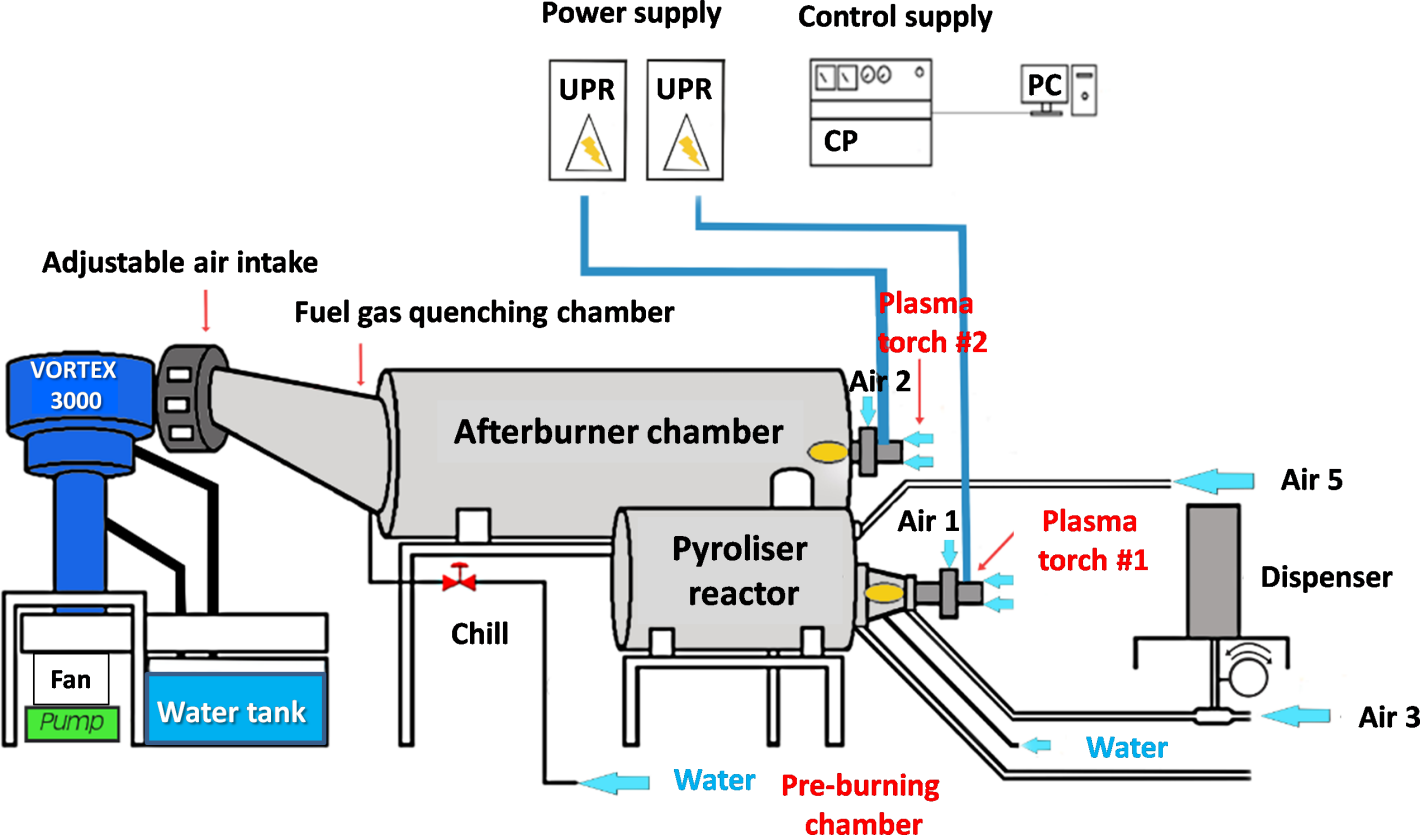
Pyrolysis/combustion plasma system.

The first step of this work consists of the reduction of volume by combustion of carbon present in fly ash. Figure 1 shows the system based on plasma arc technology used for this step. The thermal plasma system consists of two plasma-reaction chambers, one for pyrolysis and the second for final combustion. The system also contains a loading system, a working gas unit, a cooling water unit, a power supply system, and a gas cleaning system. In the two plasma-reaction chambers, the working gas is air and is injected axially into the two plasma torches at a flow rate of 45 m^3^/h. The current intensity and the voltage for the pyrolysis and combustion plasma torches are 100 A/220 V and 150 A/220 V, respectively. A hydrofilter is used for purification of the exhaust flow from mechanical impurities, sprays, vapors, and gas impurities. The feeder is filled with fly ash, and will mechanically provide for loading 25 kg/h into the plasma-reaction chamber.

**Figure 2:**
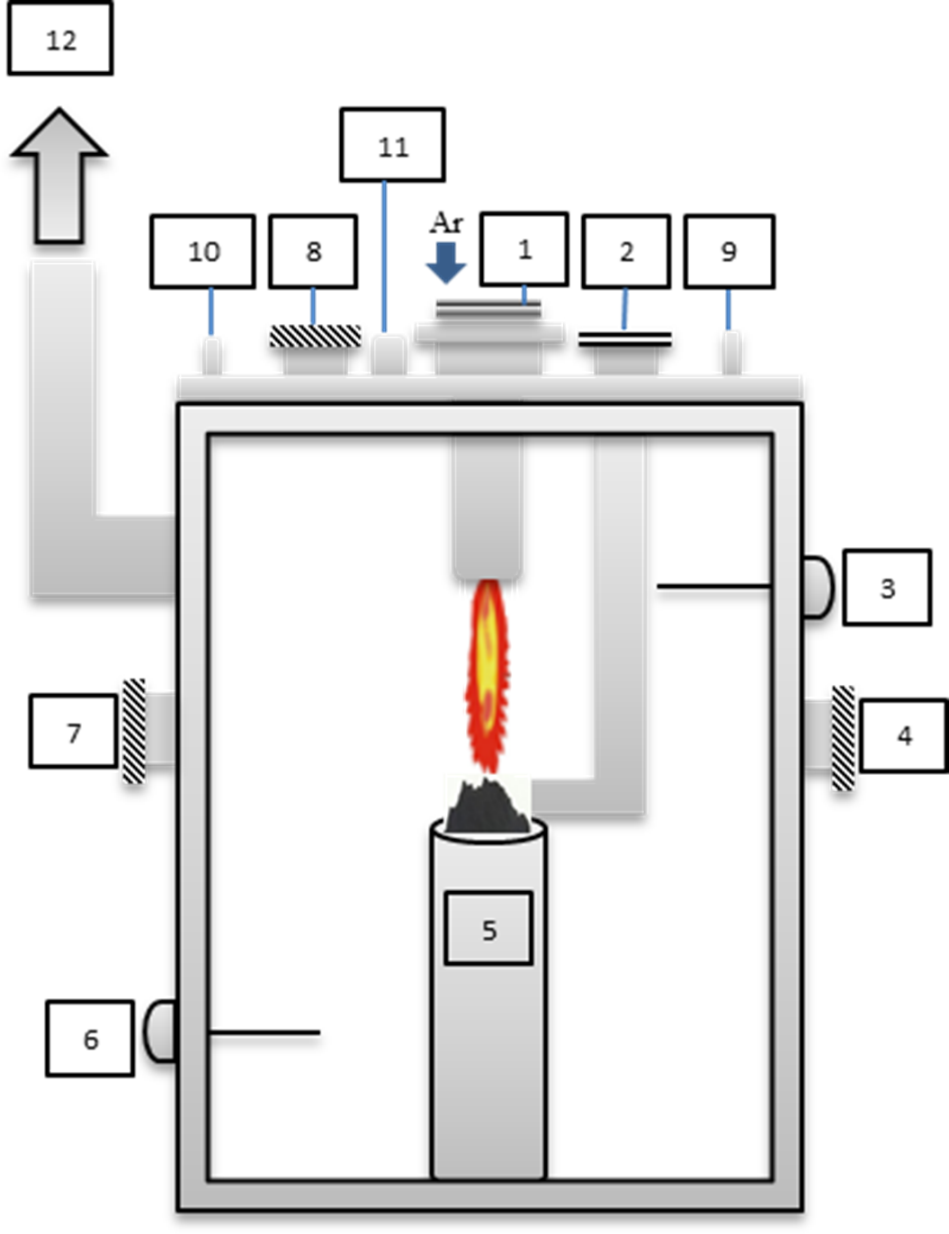
Vitrification plasma system: 1) torch, 2) feeder, 3 and 6) thermocouples, 4 and 7) windows, 5) crucible, 8) inspection window, 9) water inlet, 10) water outlet, 11) pressure sensor, 12) exhaust.

In the second step, the treated fly ash (product) was vitrified in the system shown in Figure 2. The torch was mounted above a crucible filled with the product and a glass frit (to increase the mass of Si in the product). The water-cooled stainless steel crucible is set just under the coupling zone of the plasma torches. During the experiments reported here, the current intensity is 100 A and the voltage is 220 V. In this step an inert gas (argon) is used as working gas at a flow rate of 45 m^3^/h.

### Analysis methods

The surface of the fly ash before (feed) and after treatment (product) were characterized using scanning electron microscopy (FEI INSPECT-F50-SEM, Netherlands). Chemical composition of feed and product were measured by X-ray fluorescence measurements (Shimadzu XRF-1800 Sequential X-Ray Fluorescence Spectrometer, Japan). The carbon content in the feed and the product were determined using a CHN analyzer (ELTRA CW multiphase-determinator, Germany). The concentrations of elements presents in the feed, product, and the pulverized slag were analyzed using inductively coupled plasma atomic emission spectroscopy (ICP-AES). The leaching behavior of heavy metals in the slag and fly ash was measured according to “Method 1311 Toxicity Characteristic Leaching Procedure” of the United States Environmental Protection Agency (EPA TCLP 1311). In the beginning of the leaching analysis, the amount of solids in the sample was specified. A sample that contains less than 0.5% dry solid material is considered as TCLP extract. The extraction was carried out using acetic acid as TCLP extraction fluid (20 times the weight of the solid phase) for 18 hours on an agitation tumbler (end-over-end shaking speed: 30 ± 2 rpm). The extracted fluid was tested by using the extraction fluid buffer while maintaining the pH value at 4.93. After that, the fluid was filtered through a 0.6 μm membrane filter in order to separate the TCLP extract from the solid phase. Finally, the obtained liquid was analyzed by using inductively coupled plasma mass spectroscopy (ICP-MS).

## Results and Discussion

### Characterization of fly ash

**Figure 3:**
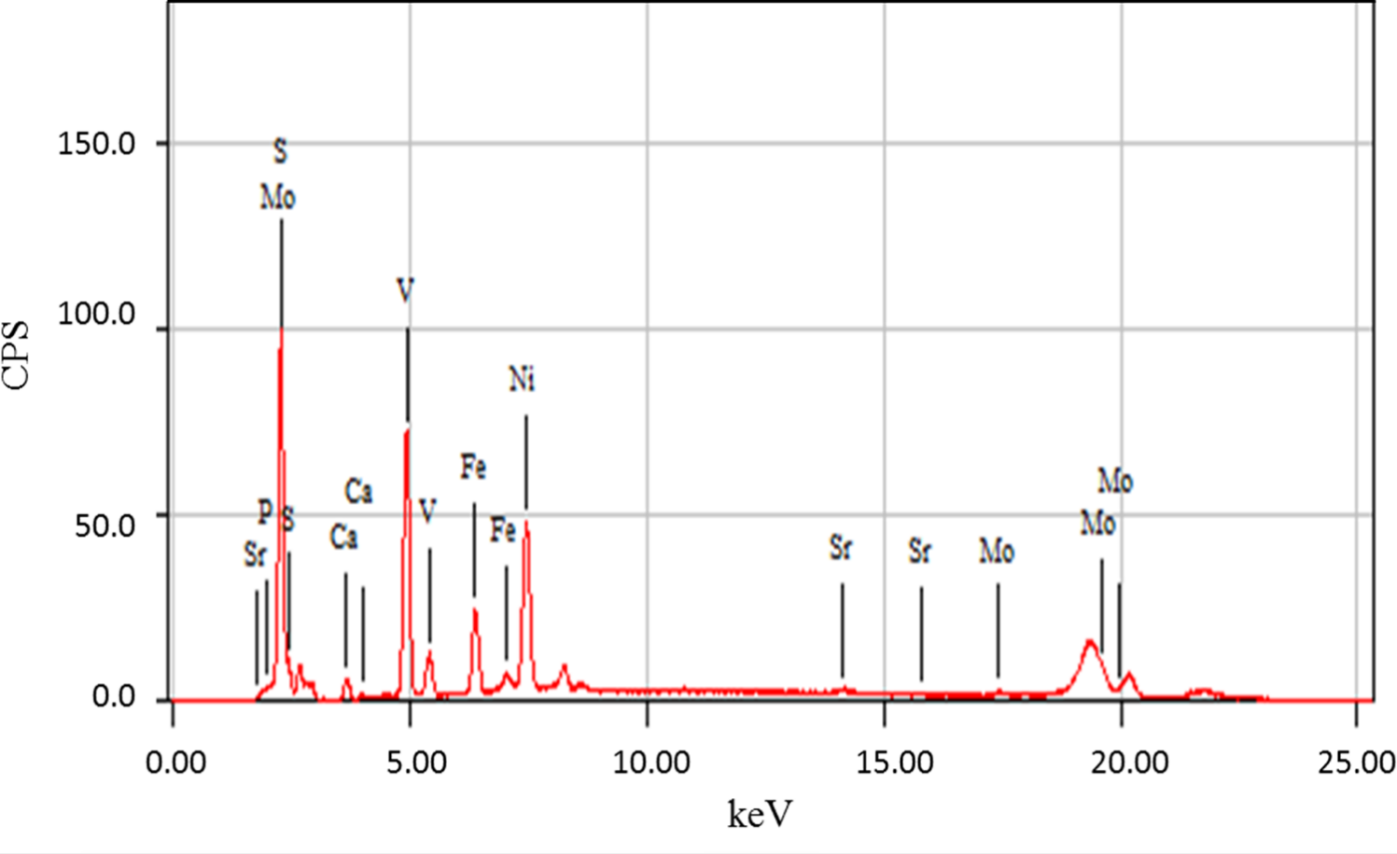
XRF analysis of feed.

The chemical composition of the feed determined with XRF is presented in Figure 3. The major constituents of the feed are NiO, Fe_2_O_3_, SO_3_, V_2_O_5_, CaO, P_2_O_5_, SrO and MoO_3_. Table 1 gives the concentration of each element present in the feed measured by ICP-AES. Many elements are not listed in Table 1 because of the very low concentration. These results confirm the XRF analysis. The major constituents (100–1000 mg/kg) in fly ash samples were V, Ca, Mg, Na, Fe, Ni, and Rh. The minor constituents (10–100 mg/kg) include Br, Si, and Al. The fly ash contains also toxic elements such as Pb, As, Zn, and Cr but with relatively low concentrations.

**Table 1:** Concentration of different elements present in feed and product (mg/kg) and measured by ICP-AES.

element	V	Ca	Al	Mg	Na	Fe	Ni	Rh	Br	Si

feed	952.8	910.4	39.8	495.3	319.5	106.1	82.9	72.4	61.6	49.6
product	910.4	106.1	49.6	319.5	72.4	952.8	495.3	39.8	39.2	36.4

element	Zn	Pb	Mo	Ba	K	As	Sr	Ti	Cr	Mn

feed	39.2	36.4	8.7	7.8	7.3	6.5	6.3	2.8	2.3	1.7
product	7.8	6.3	6.5	2.8	1.7	1.6	1.1	7.3	61.6	8.7

In addition, the fly ash samples have been analyzed for carbon, sulfur, nitrogen and hydrogen (CHN elemental analysis). Table 2 shows the results of the fly ash samples. It should be noted that fly ash is black, which indicates a high carbon content (90.81 wt %). It also contains a high amount of sulfur (6.17 wt %).

**Table 2:** Composition of feed and product determined by CHN elemental analysis.

	feed			product	
			
	wt %	atom %		wt %	atom %

carbon	90.81	95.20		91.12	95.71
oxygen	3.02	2.37		2.02	1.59
hydrogen	0.074	0.072		0.064	0.053
nitrogen	0	0		0	0
sulfur	6.17	2.42		6.86	2.70

### Combustion of fly ash

As indicated in Table 2, fly ash is mainly composed of carbon, sulfur and residue ash, whereas carbon is the dominant element of carbon black. For this reason, the first step of treatment consists of the reduction of the carbon fraction by combustion (Figure 1). In this experiment, 2.4 kg of feed were introduced in the pyrolysis chamber and only 350 g of product were collected at the exit of the combustion chamber, which amounts to a reduction of fly ash mass of 85%.

**Figure 4:**
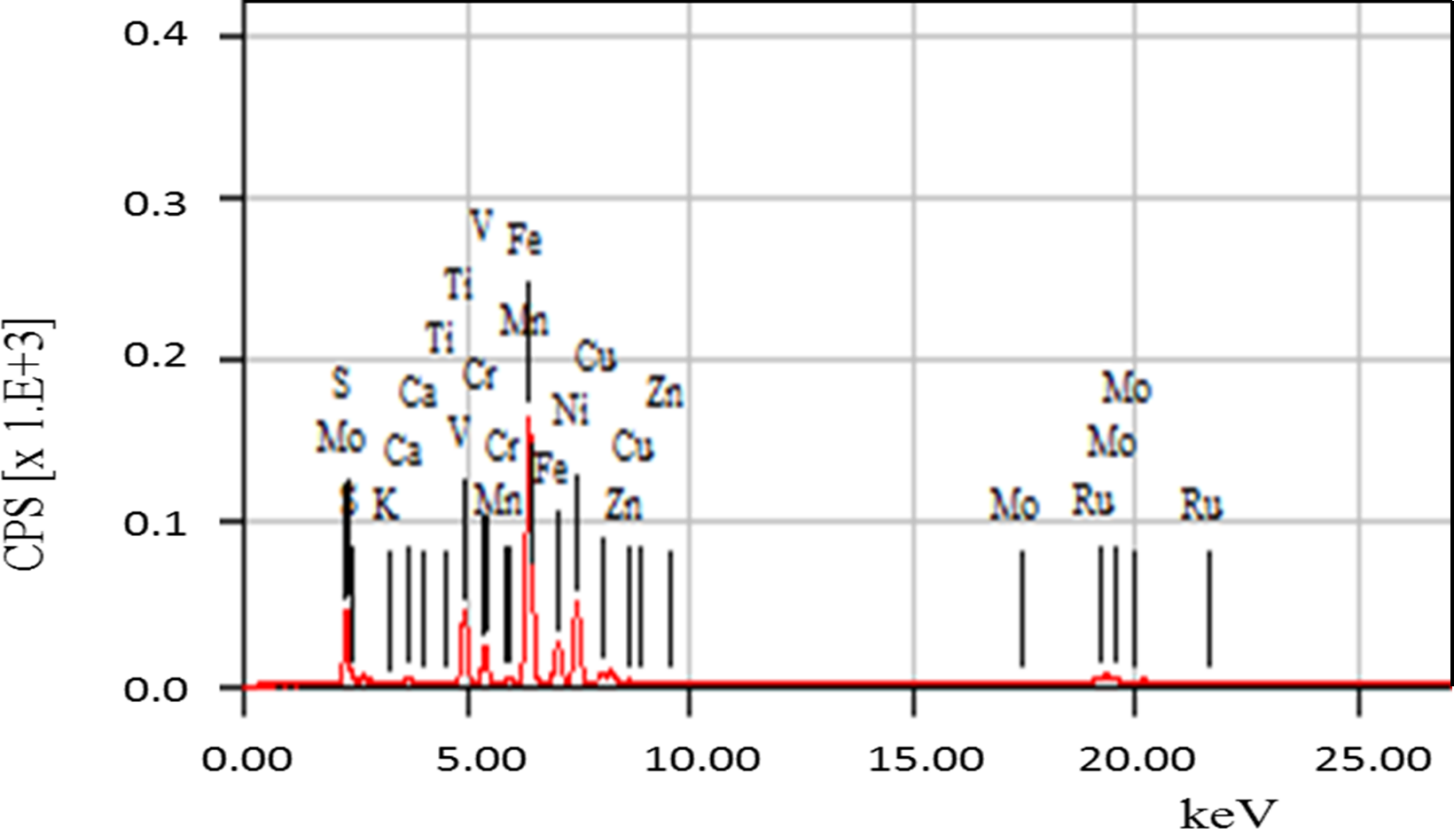
XRF analysis of product.

Figure 4 shows the chemical composition of the product obtained by XRF. This analysis shows that the major constituents of the product are the same present in the feed. Also, important contributions of K_2_O, TiO_2_, ZnO, Cr_2_O_3_, CuO, and RuO_2_ were detected. The concentrations of product elements measured by ICP-AES are summarized in Table 1.

The product has the same major and minor constituents of the feed (Table 1) and also contains toxic elements. As indicated before, fly ash has a high level of carbon content, and carbon is also the major constituent of the product (91.12 wt %, Table 2).

Table 3 gives the mass of each element present in the fly ash before and after the treatment by thermal plasma. The element masses are obtained from the concentrations presented in Table 1 and taking into consideration the ratio of fly ash mass to the product mass. The mass of each element in Table 3 is calculated according to the following equation:

[1]
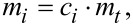


where *m_i_* is the mass of element (mg), *c_i_* is the concentration of the element (mg/kg) and *m_t_* is the mass of fly ash (kg). The mass of fly ash is 2.4 kg before processing and 0.35 kg after processing. After the combustion of fly ash the masses of the metals decrease (more than 40 % for V, and more than 90 % for Ca). The weights of Fe, Cr, and Mn increase because these metals are used as refractory material in the combustion chamber.

**Table 3:** Mass (mg) of each elements present in the fly ash before and after treatment by combustion/pyrolysis plasma. The masses were calculated from Table 1 and using Equation 1.

element	V	Ca	Mg	Na	Fe	Ni	Rh	Br	Si	Al

feed	777.46	519.93	331.661	288.61	256.01	247.12	133.46	86.161	55.359	34.422
product	455.22	53.089	159.75	36.24	476.43	247.65	19.92	19.62	18.22	24.80

element	Zn	Pb	Mo	Ba	K	As	Sr	Ti	Cr	Mn

feed	11.12	8.41	7.95	6.36	5.75	5.47	5.18	4.96	3.03	1.88
product	3.92	3.18	3.25	1.42	0.85	0.83	0.54	3.66	30.82	4.36

**Figure 5:**
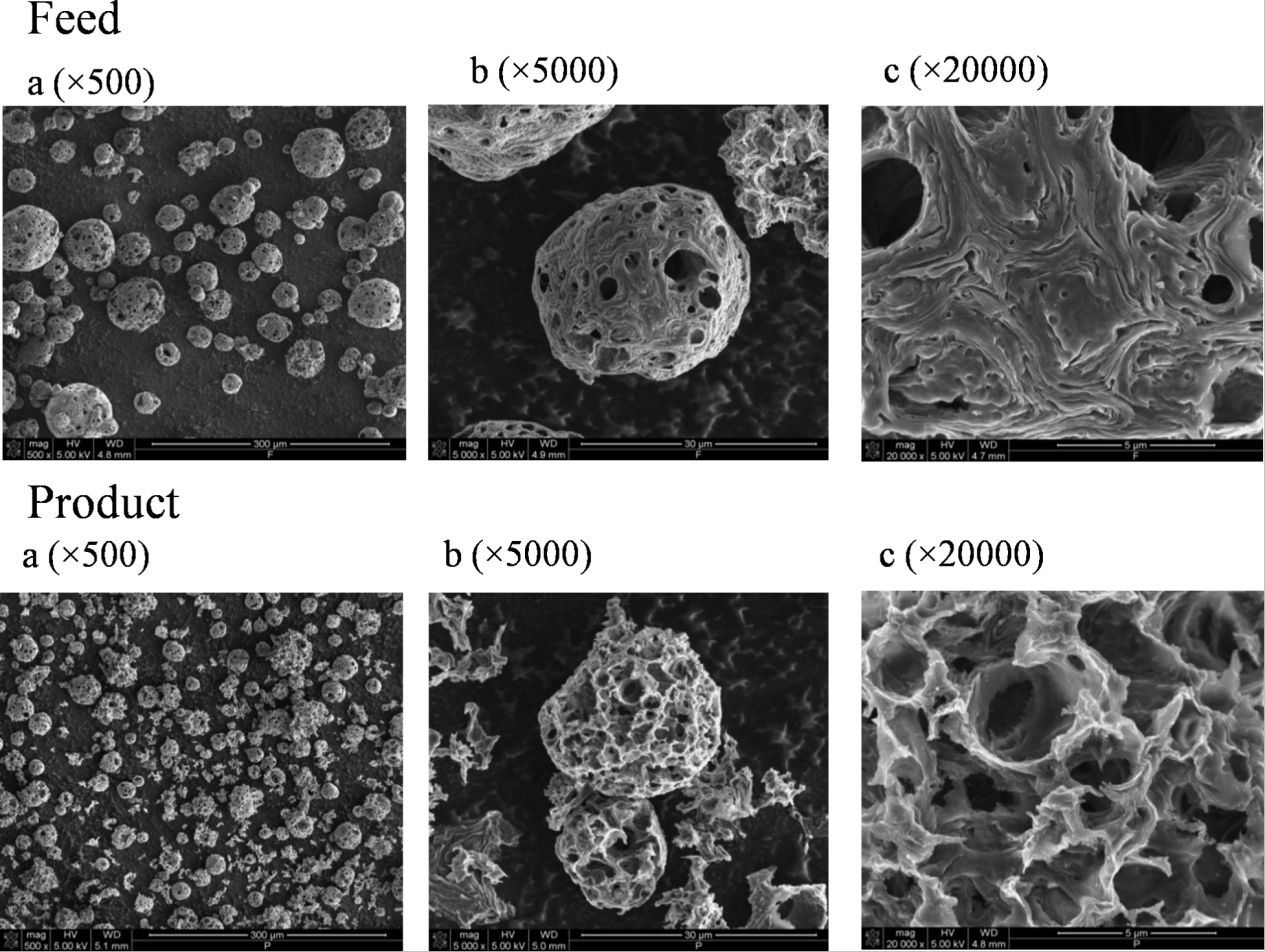
SEM images of feed and product.

SEM images of feed and product are shown in Figure 5. The fly ash has different particle sizes and the particle shapes are not regular. The carbonaceous nature of the feed and product is deduced from the porous particles as viewed in Figure 5 at 500× and 5000× magnifications. At 500× magnification, highly porous particles of diameters between 20 and 50 μm for feed and between 10 and 30 μm for product are obtained. At 5000× magnification, highly porous particles of *d*_p_ ≈ 30 μm (feed) and *d*_p_ ≈ 15 μm (product) can clearly be seen. The presence of crystalline phases is detected at 20,000× magnification at which oxides of Mg, V, Ni, Fe and S appear as plates [12–14].

### Vitrification and leaching of metals

**Figure 6:**
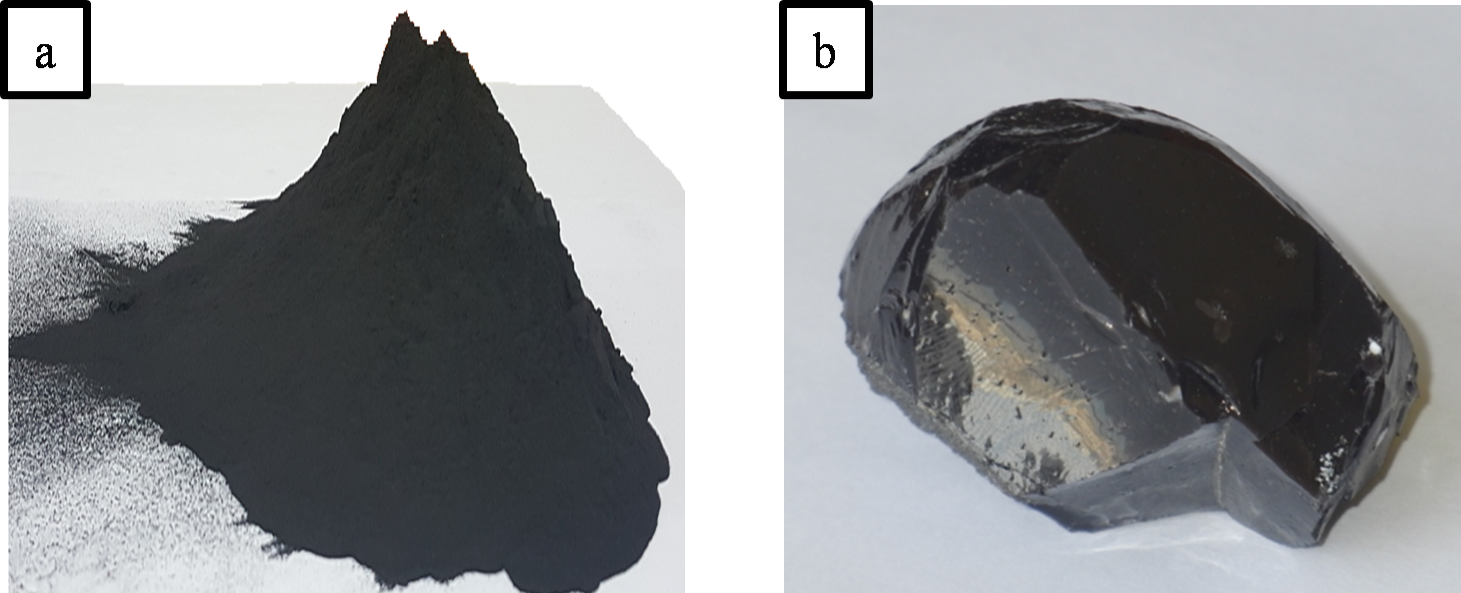
Photographs of a) fly ash and b) vitrified slag.

The product was subsequently vitrified in the system shown in Figure 2. To increase the Si content, 1 kg of glass frit was added to 1 kg of the product. Figure 6 shows a photograph of fly ash and the obtained slag. The produced slag is amorphous and glassy. The hardness and the density of the slag are 5 GPa and 1.69 g/cm^3^, respectively.

In order to evaluate the release of heavy metals under harsh environmental conditions, a prolonged leaching test (14 days) was used to investigate the leaching concentrations of heavy metals from the fly ash and the slag, and the results are shown in Table 4. It can be seen that the leaching concentrations of heavy metals from fly ash were much higher than those from slag. The concentrations of the elements present in the slag were compared with the directive of the European Union for landfill for non-toxic materials and the US EPA limits [15]. The results indicated that all the values were much lower than regulatory standard limits and suggested low leaching risk of heavy metals from the slag. So the heavy metals are confined in the silicon matrix and the slag can be disposed in landfill.

**Table 4:** Test of leaching of heavy metals from fly ash and slag.

element	release (mg·cm^−2^·day^−1^)	
	fly ash	slag

lead	0.753	0.019
chromium	0.006	0.001
zinc	2.7	0.0007
arsenic	0.004	ND

## Conclusion

Fly ash collected in exhaust treatment system of power plants using heavy fuel oils is mainly composed of carbon (90.81 wt %), sulfur (6.17 wt %) and residue ash such as V, Ca, Mg, Na, Fe, Ni, and Rh. Fly ash particles are porous, they have very irregular shapes and particle sizes from 20 to 50 μm. The treatment of the fly ash was made in two steps. In the first one, the fly ash was treated by a pyrolysis/ combustion plasma system. In this phase the weight of the ash was reduced by 85 % and the metals were partially vaporized. In the second step of treatment, the product obtained by the combustion of fly ash was vitrified in a plasma furnace. The produced slag obtained by vitrification of the product is amorphous and glassy. The leaching results indicated that all measured values were much lower than regulatory standard limits and suggested a low leaching risk of heavy metals from the slag. So the heavy metals are confined in the silicon matrix and the slag can be disposed in landfill.
